# Spotlighting zoonotic strongyloidiasis: a semi-systematic review of threadworms within baboons highlights opportunities for human infections

**DOI:** 10.1186/s40249-026-01459-0

**Published:** 2026-06-17

**Authors:** Ruth Cowlishaw, Alexandra Juhász, Lucas. J. Cunningham, E. James LaCourse, J. Russell Stothard

**Affiliations:** 1 Liverpool School of Tropical Medicine, Tropical Disease Biology, Liverpool, L3 5QA UK; 2 Institute of Medical Microbiology, Semmelweis University, 1089 Budapest, Hungary

**Keywords:** Public health, Zoonosis, *Strongyloides fuelleborni*, *Strongyloides stercoralis*, *Papio*

## Abstract

**Background:**

Both humans and non-human primates are susceptible to *Strongyloides fuelleborni*, a grossly underappreciated parasitic zoonotic threadworm across the world, in addition to the more widely reported *Strongyloides stercoralis*. This semi-systematic review sought to assess the global evidence on zoonotic strongyloidiasis in baboons, Africa’s most prolific non-human primate, to better understand the zoonotic threat these animals may pose to control strategies and public health goals.

**Methods:**

Using appropriate keyword terminology, PubMed, Scopus and Web of Science databases were searched for relevant articles, up to December 2025. Articles were then screened using inclusion and exclusion criteria, extracting relevant information for: infection prevalence, baboon species, threadworm species, infection setting and diagnostic methods. Publication content was summarised using Microsoft Excel with statistical analysis on R Studio.

**Results:**

From 1588 articles [PubMed (*n* = 307), Scopus (*n* = 678) and Web of Science (*n* = 603)], a total of 44 were summarised. Across the six species of baboons currently recognised, infection prevalence differed significantly (*P* = 0.02), *Papio cynocephalus* with highest prevalence [68.9% (IQR 38.8–82.8)] and *Papio papio* with lowest [6.5% (IQR 2.5–21.8)], noting a total absence of information for *Papio kindae*. However, over two thirds of articles did not identify threadworm infections to species level. Although not statistically significant, infection prevalence by infection setting followed an ascending order of, research organisations [13.7% (IQR 8.0–37.3)], wild populations [26.0% (IQR 15.9–37.3)] and then zoological organisations [50.0% (IQR 31.8–75.0)]. Infection dynamics (e.g. baboon sex, age) were inadequately reported, moreover detection methods infrequently used molecular methods which hampered any precise incrimination of zoonotic transmission.

**Conclusions:**

Our semi-systematic review has revealed several gaps in the global epidemiology of zoonotic strongyloidiasis which may incur real consequences for its future elimination as a public health problem. Above all, we recommend improved threadworm species identification, particularly in population-level discrimination, to better identify transmission risks into humans. Narrowing these knowledge gaps should lead to improved future control strategies for strongyloidiasis globally.

**Graphical Abstract:**

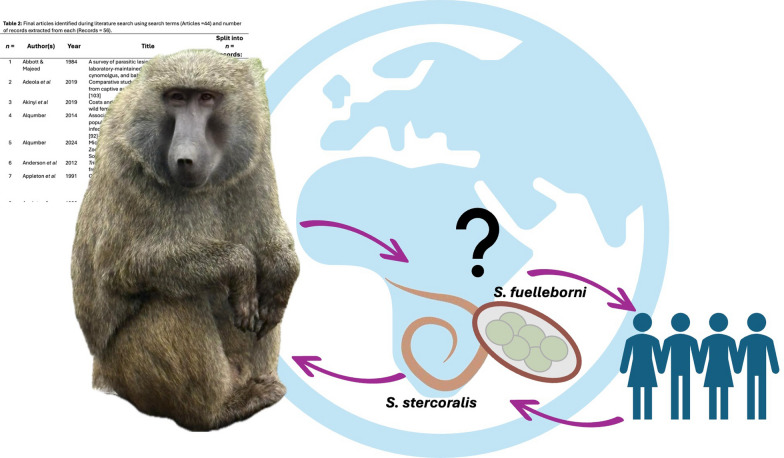

**Supplementary Information:**

The online version contains supplementary material available at 10.1186/s40249-026-01459-0.

## Background

As our closest evolutionary relatives, non-human primates (NHPs) can act as significant reservoirs of several infectious and parasitic diseases, contributing to the transmission of zoonotic pathogens into human populations around the globe [1, 2]. Due to a surge in habitat destruction and anthropogenetic changes in land use, human-NHP interactions are only increasing, resulting in an elevated risk for zoonotic transmission [3]. One source of zoonotic infections are gastrointestinal helminths, which can be commonly found in both human and NHP populations [4]. These include the soil-transmitted helminths (STHs); *Trichuris* spp., *Ascaris lumbricoides* and hookworms (*Necator americanus* and *Ancylostoma doudenale*), all of which are targeted for elimination as a public health problem by the World Health Organization (WHO) and Sustainable Development Goals [5]. *Strongyloides* spp. (threadworms) are another STH recognised for their zoonotic potential; however these worms are often overlooked and neglected in control programmes due to their more demanding requirements for specialist diagnostic techniques and anthelmintic drug treatments when compared to other STH infections [6]. However, due to its inclusion in the aforementioned WHO targets, there has been a push for improved understanding of these infections within the STH agenda, to enable more effective control strategies and fully grasp its zoonotic capabilities.

### Threadworms (*Strongyloides* spp.)

Globally, strongyloidiasis remains of major veterinary and public health concern. It is estimated that 600 million people are infected worldwide (a mean global prevalence of 8.1%), however there is no global estimate in NHPs [7]. Of the more than 50 species within the *Strongyloides* genus, just two are thought capable of causing zoonosis, infecting both humans and NHPs: *Strongyloides stercoralis* and *Strongyloides fuelleborni* [8, 9]. Although the vast majority of human infections are believed to be caused by *S. stercoralis*, there is now a growing appreciation for the role of *S. fuelleborni* in human infections [10]. Through the use of modern genotyping methods, *S. fuelleborni* infections have already been linked between humans and the following NHPs: chimpanzees, yellow baboons and southern pig-tailed macaques, co-habiting within the same environments [11, 12]. Baboons are of particular interest here as unlike other NHPs, they are susceptible to both *S. stercoralis* and *S. fuelleborni*, with the potential for concurrent infections of the two [13–15]. Concerningly, infection with *S. stercoralis* can have fatal consequences in all primates, due to its ability to autoinfect, leading to hyper-infection syndrome/disseminated strongyloidiasis [16–18]. As such, care should be taken to control the spread of infection and identify at risk populations. In contrast, there are no current reports of *S. fuelleborni* causing severe disease in either humans or NHPs with infections generally thought to be self-limiting and asymptomatic, however mild abdominal symptoms have been documented [19]. Although the previously identified sub-species *S. fuelleborni kellyi* was believed to cause swollen belly syndrome (SBS) in children in Papua New Guinea, it has now been demonstrated that *Strongyloides* infections found here can be mainly attributed to the Asia–Pacific subspecies of *S. fuelleborni fuelleborni* with incidences of SBS now being assigned to a newly identified species more closely associated with *Strongyloides ransomi* [20].

**Fig. 1:**
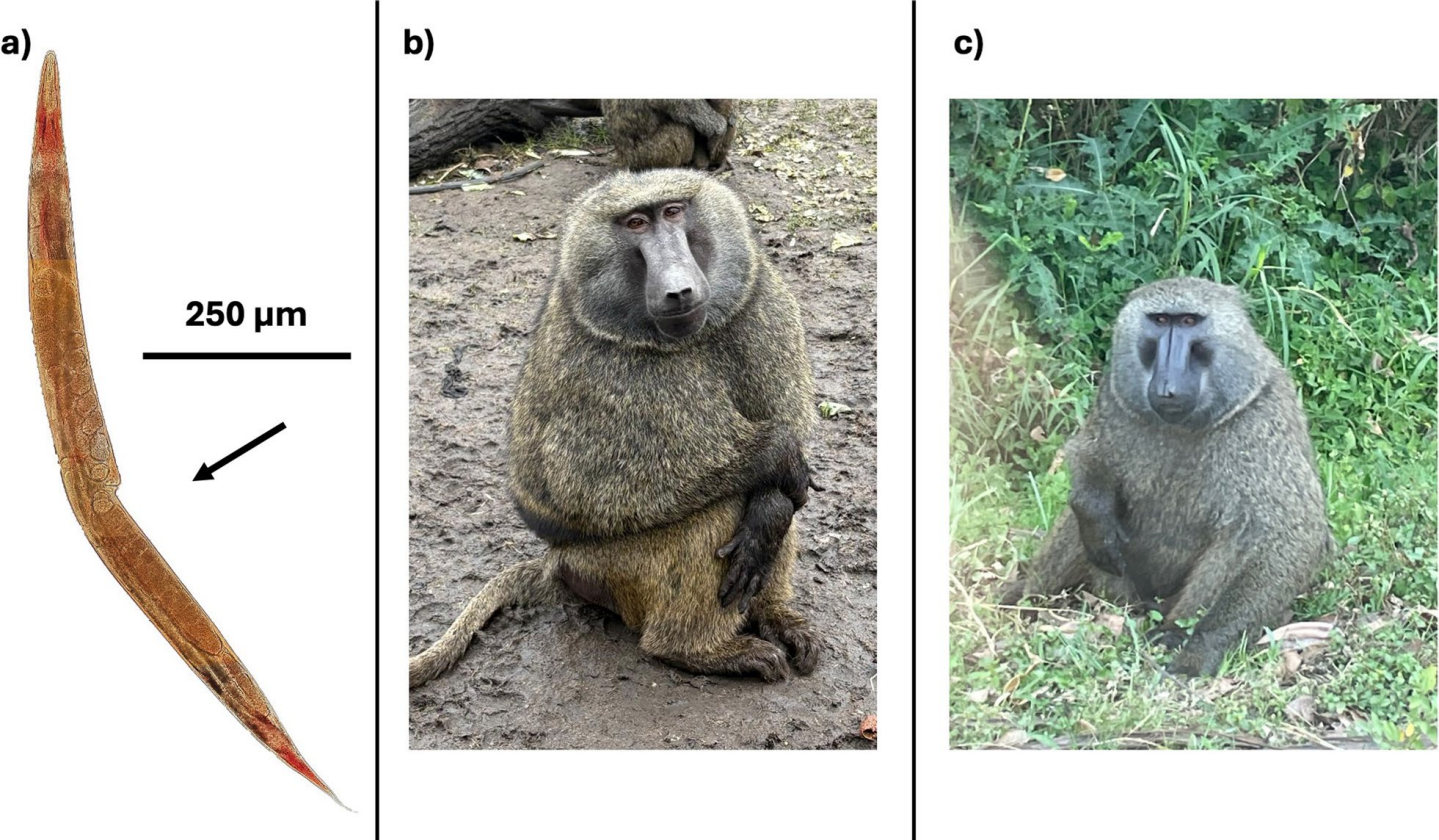
**a** Lugol’s iodine stained *Strongyloides f. fuelleborni* adult female under light microscopy. Arrow marks its characteristic vulval region that differentiates it from *S. stercoralis*. **b** Adult male baboon of mixed ancestry from Knowsley Safari, Prescott, UK. **c** Adult male olive baboon from Western Uganda

In terms of its life cycle, *Strongyloides* spp. differ from many of their parasitic-helminth cousins by having both parasitic and non-parasitic developmental routes. Eggs are deposited in faeces by parasitic female worms residing in the small intestine, where in the case of *S. stercoralis*, they hatch into L1 larvae before exiting the host [21]. Defaecation by an infected host results in the contamination of the environment, after which larvae develop through three larval stages. If conditions are favourable, L1 larvae can either develop into free-living non-parasitic male and female adult worms (Fig. 1a) to undergo one generation of sexual reproduction or alternatively develop into infective L3 larvae. Once a permissible host is detected, infective larvae burrow through the skin or mucous membranes, migrating through the respiratory system, eventually developing into adults in the bowel [22]. In humans, strongyloidiasis is primarily treated by a single dose of ivermectin (200 μg/kg) or short course of albendazole, however the latter is significantly less effective [23]. Concerningly, the current WHO guidelines for strongyloidiasis control fails to mention *S. fuelleborni* entirely, demonstrating a global oversight for this species and lack of specific treatment knowledge [24]. For example, there are currently no formal guidelines on the treatment of NHPs, yet successful treatments with ivermectin have been used on several primate species in captivity [25–27].

The genetic diversity and host specificity of both *S. stercoralis* and *S. fuelleborni* are still incompletely understood. In terms of *S. stercoralis*, it is believed the species separates into two distinct evolutionary clades, one primarily infecting primates and the other domestic dogs [19, 28]. Barret and Sapp [29] however believe this an oversimplification and propose seven distinct clusters instead, with varying degrees of host preference. By contrast, the arguments pertaining to *S. fuelleborni* are currently based around a newly appreciated geographical split between an Asian and African clade. Multiple studies have now demonstrated strong evidence of this division, with demonstration that *S. fuelleborni* may be more geographically diverse than once thought [30, 31].

### Baboons (*Papio* spp.)

All extant species of baboons belong to the genus *Papio*, which is placed within the Papionini tribe of Old-World monkeys [1]. This, amongst others includes macaques, mandrills, drills and mangabeys. Although baboon populations are known to interbreed in hybrid zones and captivity (Fig. 1b), they have been classified into six separate species due to their distinctive morphological, behavioural and social patterns [32–36]. These six species are: *Papio anubis* (Olive) (Fig. 1c), *P. cynocephalus* (Yellow), *P. ursinus* (Chacma), *P. papio* (Guinea), *P. hamadryas* (Hamadryas) and *P. kindae* (Kinda). Each has its own natural geographical range, collectively spanning much of sub-Saharan Africa and the South-West edge of the Arabian Peninsula as shown in Table 1 [34]. Five out of the six species of baboons are classified as “of least concern” by the International Union for Conservation of Nature’s Red List, however populations of guinea baboons have been reducing over many years, leading to their “nearly threatened” classification [37].

**Table 1:** Geographical distributions for all six baboon species [34]

Common name	*Papio* species	Distribution
Olive	*P. anubis*	Large parts of northern sub-Saharan Africa
Yellow	*P. cynocephalus*	Large parts of Eastern Africa
Guinea	*P. papio*	West Africa (Sierra Leonne to Mauritania)
Chacma	*P. ursinus*	Southern Africa
Hamadryas	*P. hamadryas*	Northwest Africa and Arabian Peninsula
Kinda	*P. kindae*	Western Africa (Zambia, Angola, Democratic Republic of Congo)

As Africa’s most prolific NHP, baboons are highly adaptable, able to live in a wide range of ecological environments [35]. Unlike many other primates, they have shown a strong ability to thrive in areas of high anthropogenic change such as urbanisation and tourist camps, scavenging human food sources; especially through dustbin and crop raiding [38–40]. As such, it is not unusual for baboons to come into frequent contact with humans, resulting in cross-species conflict and leads to health issues in baboons such as obesity and poor dental health [41, 42]. Wild baboon populations are not however the only potential route of infection into human populations. Baboons are a popular addition to many zoological institutions, as well as being used in experimental facilities and are known to carry many zoonotic STH infections [43–45]. As keepers/handlers have regular contact with baboon waste, for example faeces, it is important not to overlook this population and the significance it could play in occupational health [46, 47].

Due to their relatively large size and terrestrial daytime lifestyle, baboons are easily followed through environments and have commonly been the focus of many behavioural and phylogenetic studies [48–51]. However, it is this predominately terrestrial life that leads to increased contact time with contaminated soils and food sources, potentially predisposing them to STH infections, including strongyloidiasis. This, together with their extensive distribution and high anthropogenic exposure makes them an ideal model for investigating the zoonotic capabilities of NHPs within the STH agenda. Therefore, this review aimed to conduct an in-depth semi-systematic review to explore the prevalence of strongyloidiasis within baboons, highlighting both parasite (*Strongyloides*) and host (*Papio*) species, alongside global distribution, infection setting and diagnostic methodology to gain further understanding of this underappreciated zoonotic infection.

## Methods

### Search strategy

Relevant literature was found by searching key terms across three databases: PubMed, Scopus and Web of Science. During the original search “baboon/*Papio*” and “Strongyloid*/threadworm*” were utilised to search titles, abstracts and keywords. As the name of both specific host and parasite may not always be given in these areas the search was also expanded to include more overarching terms such as “primate, gastro-intestinal, soil transmitted helminth, helminth, STH” and all their derivatives. Duplicated records were identified and removed manually using EndNote21 (Clarivate, Philadelphia, PA, USA) and then again manually analysed to remove articles clearly concerned with non-*Strongyloides* or non-*Papio* species. Papers were then manually screened by the lead author in their entirety relating to the below inclusion/exclusion criteria.

### Inclusion and exclusion criteria

Texts in their entirety were assessed to determine suitability for inclusion into further analysis. The criteria for inclusion were as follows: (1) the text must in some way be part of a parasitological study, (2) there must be a clear link between *Papio* and *Strongyloides*. Articles were excluded if they were not published in English or *Strongyloides* data could not be linked to *Papio* specifically. Articles were then manually analysed for relevance and key findings summarised after quality assessment. All searched data were handled using EndNote21 (Clarivate, Philadelphia, PA, USA).

### Evidence analysis and quality assessment

Data were extracted from relevant studies, covering the following: date of publication, study location, *Strongyloides* and *Papio* species, *Strongyloides* prevalence, diagnostic methods, infection setting, study length and mentions of One Health. Descriptive analysis was undertaken by formatting data in Microsoft Excel (Microsoft Corp., Redmond, WA, USA) solely by the lead author. Quality assessment of reviewed literature was carried out using the Critical Appraisal Skills Programme (CASP) checklist for cross-sectional studies as this best suited the majority of literature found [52].

After data summarisation, it was noted that infections in *P. kindae* were absent from the records, therefore a secondary, more broad literature search using the search terms “*Papio kindae*” OR “kinda baboon*” only was carried out. This resulted in a total of 79 articles (PubMed = 14, Web of Science = 46, Scopus = 19), however after a manual screen, none successfully passed our inclusion criteria.

As several articles investigated multiple study sites or baboon species, prevalence and *Strongyloides* species data were treated separately where possible. Three groups were created to classify infection setting: Zoological Organisations, Research Organisations and Wild populations. These data were compiled using Microsoft Excel (Office 2024) and then statistical analysis was undertaken using R Studio [53]. Overall, prevalence was found to be non-normally distributed, therefore non-parametric tests (Kruskal-Wallis) were applied, with post-hoc testing via the Dunn test [54]. Results are presented as median percentage (%) values along with the inter-quartile range (IQR).

## Results and discussion

**Fig. 2:**
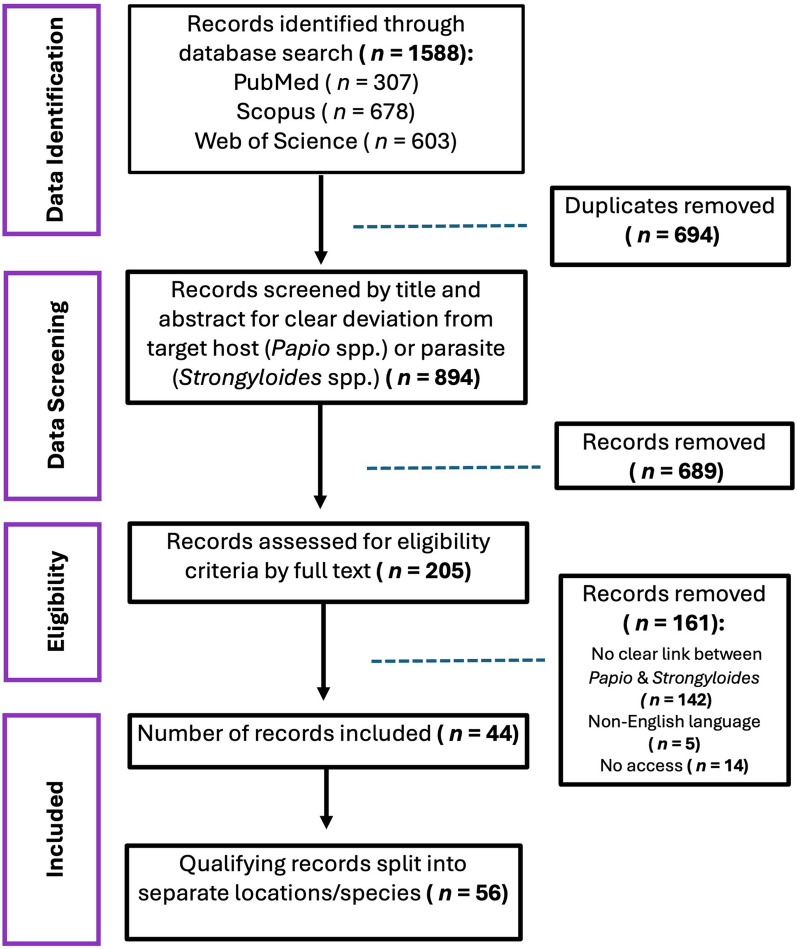
Flow diagram of the search for suitable literature

Excluding the additional search for *P. kindae* references, a total of 1588 records were initially identified for the purpose of this review using key search terms over three databases. Initially 694 records were then removed due to duplication and then a further 850 were excluded having not met the stipulated review criteria: firstly, using titles and abstracts and then full text assessments. The high number of rejections is a result of including non-specific search terms such as “gastro-intestinal” and “primates”. Finally, 44 articles remained and were included in the final review (Fig. 2). As multiple studies included data from various locations within a country or investigated/recorded different host/parasite species, these were separated out resulting in a total of 56 records for inspection (Table 2). Figure 2 demonstrates the process taken to obtain relevant review material. Here we show and discuss our findings in terms of host and parasite species, global distribution, infection setting and diagnostics.

**Table 2:** Final articles identified during literature search using search terms (Articles = 44) and number of records extracted from each (Records = 56)

*n* =	Author(s)	Year	Title	Split into *n* = records:
1	Abbott and Majeed	1984	A survey of parasitic lesions in wild-caught, laboratory-maintained primates: (Rhesus, Cynomolgus, and Baboon) [55]	1
2	Adeola et al	2019	Comparative study of gastrointestinal parasites from captive and wild olive baboon (*Papio anubis)* [56]	2
3	Akinyi et al	2019	Costs and drivers of helminth parasite infection in wild female baboons [57]	1
4	Alqumber	2014	Association between *Papio hamadryas* populations and human gastrointestinal infectious diseases in southwestern Saudi Arabia. [58]	1
5	Alqumber	2024	Microbiological ecological surveillance of zoonotic pathogens from Hamadryas baboons in Southwestern Saudi Arabia [59]	3
6	Anderson et al	2012	*Trichuris* sp. and *Strongyloides* sp. infections in a free-ranging baboon colony [43]	1
7	Appleton et al	1991	Gastro-intestinal helminth parasites of the chacma baboon, *Papio cynocephalus ursinus*, from the coastal lowlands of Zululand, South Africa [14]	2
8	Appleton and Henzi	1993	Environmental correlates of gastrointestinal parasitism in montane and lowland baboons in Natal, South Africa [60]	1
9	Appleton and Brain	1995	Gastro-intestinal parasites of *Papio cynocephalus ursinus* living in the central Namib desert, Namibia [61]	1
10	Barelli et al	2020	Loss of protozoan and metazoan intestinal symbiont biodiversity in wild primates living in unprotected forests [62]	1
11	Barelli et al	2021	Interactions between parasitic helminths and gut microbiota in wild tropical primates from intact and fragmented habitats [63]	1
12	Bezjian et al	2008	Coprologic evidence of gastrointestinal helminths of forest baboons, *Papio anubis*, in Kibale National Park, Uganda [64]	1
13	Bradbury et al	2025	Intestinal parasite infection in non-human primates from The Gambia, West Africa, and their relationship to human activity [65]	1
14	Ebbert et al	2013	Community composition, correlations among taxa, prevalence, and richness in gastrointestinal parasites of baboons in Senegal, West Africa [66]	2
15	Eley et al	1989	Nutrition, body condition, activity patterns, and parasitism of free‐ranging troops of olive baboons (*Papio anubis*) in Kenya [67]	2
16	Fagiolini et al	2010	Gastrointestinal parasites in mammals of two Italian zoological gardens [68]	1
17	Fredrick et al	2021	*Schistosoma mansoni* and soil transmtted helminths in olive baboons and potential zoonosis [69]	2
18	Goldsmid	1974	The use of mebendazole as a broad spectrum anthelmintic in Rhodesia [13]	1
19	Habig et al	2021	Predictors of helminth parasite infection in female chacma baboons (*Papio ursinus*) [70]	1
20	Hahn et al	2003	Gastrointestinal parasites in free-ranging Kenyan baboons (*Papio cynocephalus* and *P. anubis*) [71]	2
21	Hope et al	2004	Parasitic health of olive baboons in Bwindi Impenetrable National Park, Uganda [72]	1
22	Howells et al	2011	Patterns of gastro-intestinal parasites and commensals as an index of population and ecosystem health: the case of sympatric western chimpanzees (*Pan troglodytes verus*) and guinea baboons (*Papio hamadryas papio*) at Fongoli, Senegal [73]	1
23	Juhàsz et al	2023	Gastrointestinal parasites in captive olive baboons in a UK safari park [44]	1
24	Kebede et al	2018	*Schistosoma mansoni* infection in human and nonhuman primates in selected areas of Oromia Regional State, Ethiopia [74]	1
25	Kooriyama et al	2012	Parasitology of five primates in Mahale Mountains National Park, Tanzania [75]	1
26	Labri et al	2020	Zoonotic gastrointestinal parasites of baboons (*Papio anubis*) in the Shai Hill Reserve in Ghana [76]	1
27	Labri et al	2021	Distribution of intestinal parasites of baboons (*Papio anubis*) and warthogs (*Phacochoerus aethiopicus*) at the Mole National Park, Ghana [77]	1
28	Legesse and Erko	2004	Zoonotic intestinal parasites in *Papio anubis* (baboon) and *Cercopithecus aethiops* (vervet) from four localities in Ethiopia [78]	1
29	Mafuyai et al	2013	Baboons as potential reservoirs of zoonotic gastrointestinal parasite infections at Yankari National Park, Nigeria [79]	1
30	Mason et al	2022	Association of human disturbance and gastrointestinal parasite infection of yellow baboons in western Tanzania [80]	1
31	Mbuthia et al	2021	Potentially zoonotic gastrointestinal nematodes co-infecting free ranging non-human primates in Kenyan urban centres [81]	1
32	McGrew et al	1989	Intestinal parasites of sympatric *Pan troglodytes* and *Papio* spp. at two sites: Gombe (Tanzania) and Mt. Assirik (Senegal) [82]	2
33	Müller-Graf et al	1996	Intestinal parasite burden in five troops of olive baboons (*Papio cynocephalus anubis*) in Gombe Stream National Park, Tanzania [83]	1
34	Munene et al	1998	Helminth and protozoan gastrointestinal tract parasites in captive and wild-trapped African non-human primates [84]	2
35	Obanda et al	2019	Infection dynamics of gastrointestinal helminths in sympatric non-human primates, livestock and wild ruminants in Kenya [15]	*Prevalence not provided but included in distribution analysis
36	Ocaido et al	2003	Gastrointestinal parasites of baboons (*Papio anubis*) interacting with humans in West Bugwe Forest Reserve, Uganda [85]	3
37	Ofori et al	2024	Sharing without caring: High prevalence and similarity of potentially zoonotic gastrointestinal helminths in two sympatric nonhuman primates in a tropical resource reserve [4]	1
38	Owen and Casillo	1973	A preliminary survey of the nematode parasites of some imported old-world monkeys [86]	2
39	Pebsworth et al	2012	Parasite transmission risk from geophagic and foraging behavior in Chacma Baboons [87]	1
40	Pettifer	1984	The helminth fauna of the digestive tracts of chacma baboons, *Papio ursinus*, from different localities in the Transvaal [88]	1
41	Reichard et al	2017	Pilot study to assess the efficacy of ivermectin and fenbendazole for treating captive-born olive baboons (*Papio anubis*) coinfected with *Strongyloides fülleborni* and *Trichuris trichiura* [89]	1
42	Ryan et al	2012	A survey of gastrointestinal parasites of olive baboons (*Papio anubis*) in human settlement areas of Mole National Park, Ghana [90]	1
43	Shemshadi et al	2015	Prevalence and intensity of intestinal helminths in carnivores and primates at Vakilabad Zoo in Mashhad, Iran [91]	1
44	Weyher and Ross	2006	Gastrointestinal parasites in crop raiding and wild foraging *Papio anubis* in Nigeria [92]	1
Total: 44				Total: 56

### Infection prevalence by baboon species

The olive baboon (*P. anubis*) was by far the most studied baboon species in this area; not surprising given its wider distribution across the African continent. Conversely, *P. kindae* was completely absent from any mention of *Strongyloides*. This is likely due to the distinct lack of parasitological investigations altogether in the species, rather than a complete absence of infections in these populations as demonstrated by the additional and directed literature search. *Papio kindae* inhabits large parts of West Africa, therefore considerable knowledge gaps in this area could have consequences to infection control in the future. Baboons are likely to express varying behaviours in different localities, so it is therefore important to gather risk data at the local level [93].

**Fig. 3:**
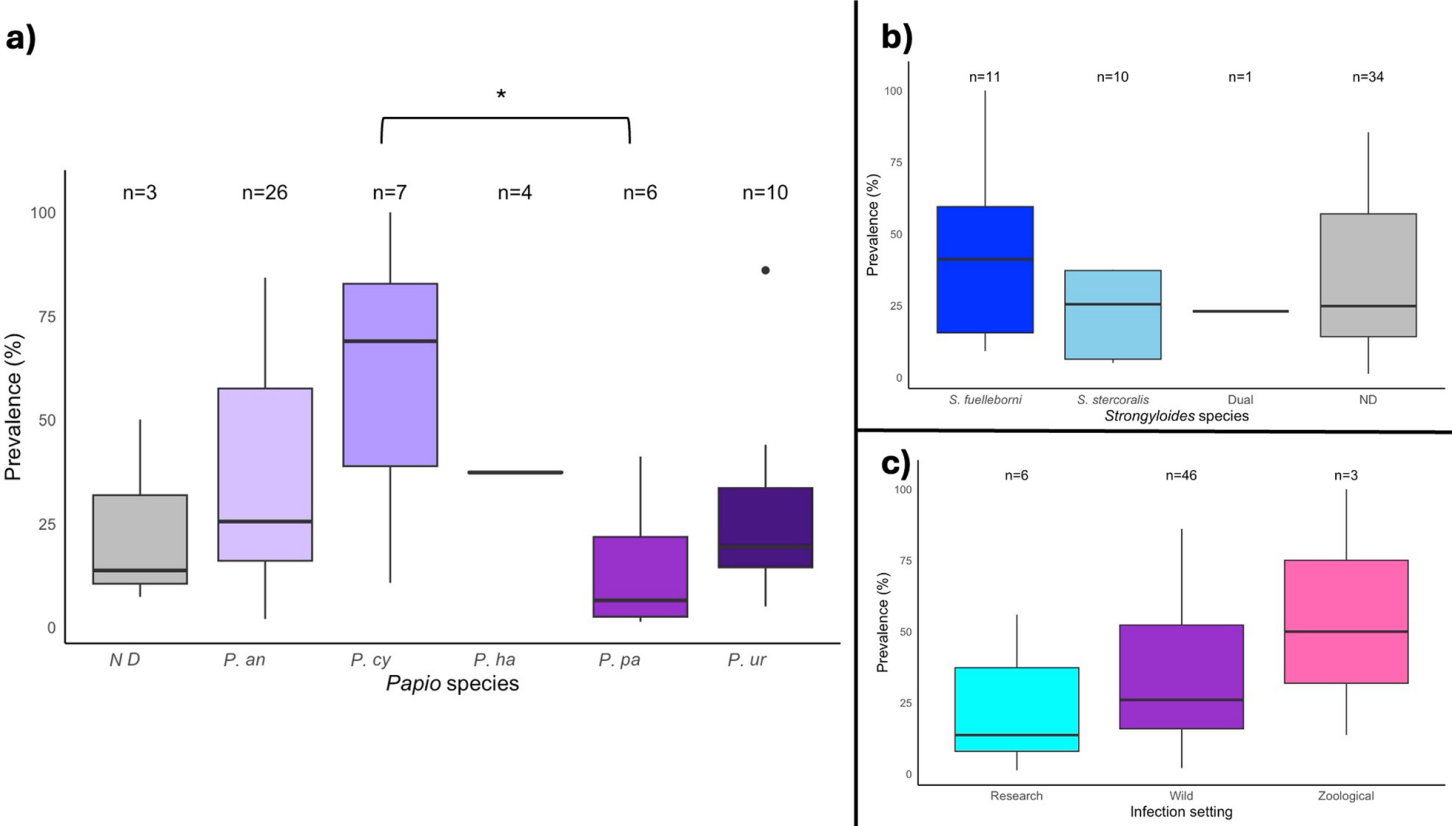
Box plots of *Strongyloides* prevalence in baboons by **a** *Papio* species, **b** *Strongyloides* species and **c** infection setting. Data were summarized are from split data list (*n* = 56). Number of records in each category is displayed above

On analysis, a significant difference in parasite prevalence was observed between *Papio* species (Kruskal-Wallis test: χ^2^ = 11.05, df = 5, *P* = 0.05). After pairwise comparisons using the Dunn’s test, a significant increase in parasite prevalence was found in *P. cynocephalus* when compared to *P. papio* (*P* = 0.02), whilst all other comparisons were non-significant (Fig. 3a). The sample sizes of all baboon species bar *P. anubis* were low, therefore an increase in sample size would improve overall statistical power here. As different species of baboons have different home ranges, it is challenging to make direct comparisons between species as many factors such as genetics, behaviour or habitat could be affecting *Strongyloides* prevalence. For this review attempts were made to correlate *Papio* species with *Strongyloides* species, however due to the high number of non-specified infections, this was not possible.

### Infection prevalence by *Strongyloides* species

Both *S. stercoralis* and *S. fuelleborni* are capable infecting baboons, however in 61% of cases here, *Strongyloides* species was not specified precisely. When specified, *S. fuelleborni* was the most prevalent species (*n* = 11) closely followed by *S. stercoralis* (*n* = 10) and then dual infections (*n* = 1). In terms of dual infection, it was not possible to decipher whether these infections have occurred concurrently or are from different animals from the reported data [14]. However, the two species are clearly circulating in a similar environment, giving rise to questions on cross species interactions in terms of both pathogenicity and genomics. *Strongyloides* species did not appear to significantly affect infection prevalence (Kruskal–Wallis test: χ^2^ = 2.27, df = 3, *P* = 0.52), even when studies with non-defined species were removed (Kruskal–Wallis test: χ^2^ = 2.82, df = 2, *P* = 0.24), as seen in Fig. 3b. There is very little information on infection dynamics for these species in primates available for discussion, therefore we can only assume that both *S. stercoralis* and *S. fuelleborni* have similar infection rates within baboon populations. The overall lack of threadworm speciation is discouraging, especially as species identification is crucial for appreciating potential disease pathology in humans and NHPs alike. Chosen diagnostic methods are likely culpable here as *Strongyloides* larvae and eggs are difficult to differentiate from other strongylid nematodes using microscopy alone without adequate training. This not only incurs the danger of over simplifying parasite diversity but also presents challenges for mapping *Strongyloides* species distribution accurately, as recently demonstrated by Cunningham et al. [10].

### Distribution and infection setting

**Fig. 4:**
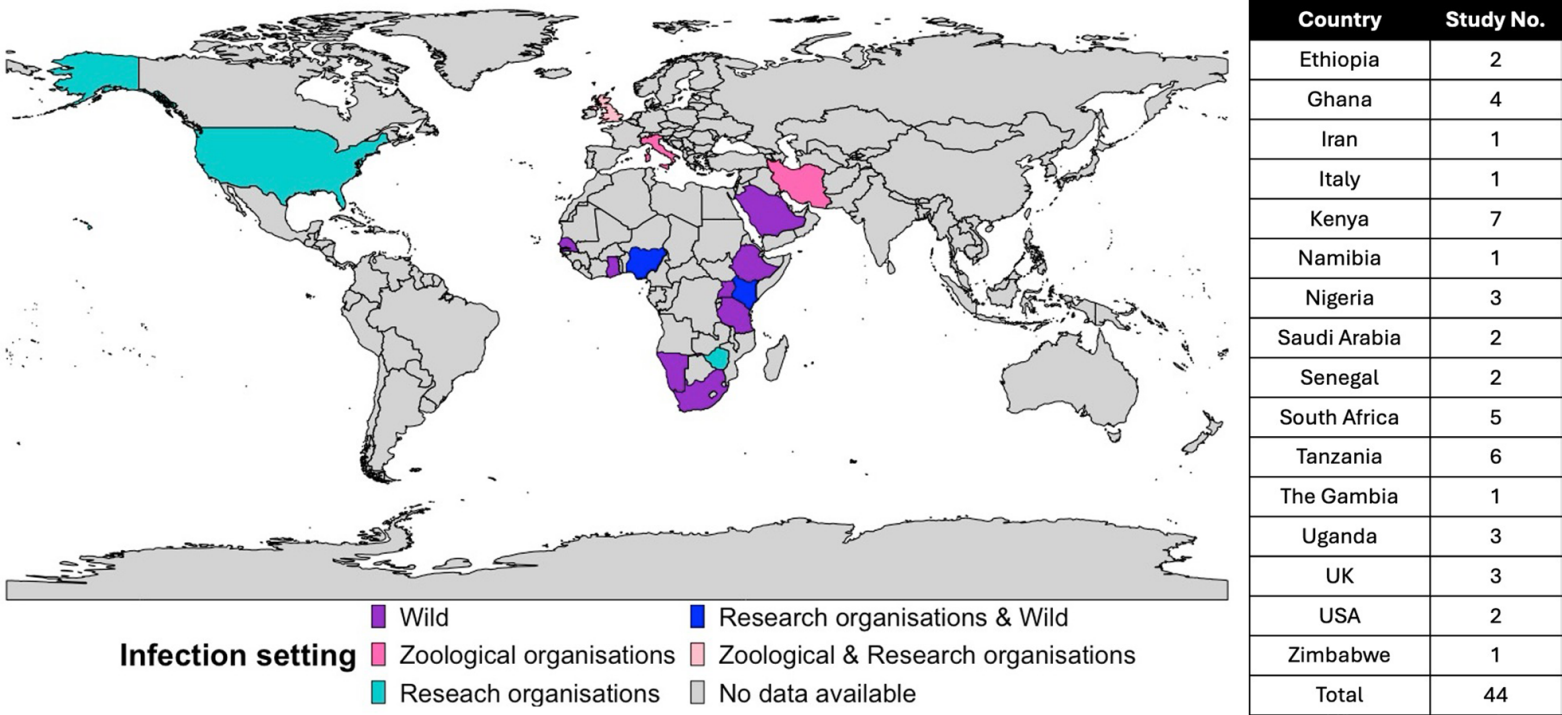
Map showing global distribution of *Strongyloides* infections within *Papio* spp. by infection setting from original (*n* = 44) articles. The number of records from each country is outlined in the table

From information gathered during this review, the identification of *Strongyloides* species within baboons has been recorded in a total of 16 countries, predominantly focussing on wild populations in Africa, Fig. 4. Although the baboon’s natural distribution mainly falls within Africa, this collection of studies is unlikely to represent the true extent of infections within these animals, particularly when thinking of the distribution of animals within zoological and research organisations. Adding to this, many investigations into wild populations tend to take place within national parks, which may not fully present the full picture of zoonotic potential, due to decreased opportunity for cross-species contact than in areas of higher anthropogenic change [72, 79, 94]. Together, this presents large areas of naivete and lost opportunities to study the dynamics of the infection within these primates.

When comparing *Strongyloides* prevalence by infection setting (wild, research organisations and zoological organisations), no significant difference was found (Kruskal-Wallis test: χ^2^ = 2.30, df = 2, *P* = 0.31). These results however should be taken with caution as the number of studies reporting prevalence in research and zoological populations was low (*n* = 6 and 3 respectively), compared to those reported from wild populations (*n* = 47). This likely skewed the data and underpowered the analysis of zoological and research groups. However these findings do align with Munene et al., who showed no significant differences between the helminth parasites of captive and wild caught baboons in Kenya [84]. Although not significant, differences were seen between infection settings in the current study; zoological populations had a higher overall median prevalence [50.0% (31.8–75.0)], than wild [26.0% (15.9–52.2)] and research-based organisations [13.7% (7.9–37.3)], as seen in Fig. 3c.

Less than a quarter of studies included in this review investigated captive baboon populations. Although several studies in the original data search noted both the discovery of *Strongyloides* and baboons in their cohort, primate data covering multiple species was often combined making it difficult to extract baboon specific data [95, 96]. Captive animals are an excellent way to study specific infection transmission due to their accessibility and semi-controlled environments. However, these populations are often subject to periods of anthelmintic treatment, live in restricted ranges (increasing chances of infection) and are fed by their keepers, creating disparities when compared to natural infection dynamics, which should be considered.

### Infection dynamics within baboons

Many factors e.g. seasonality, behaviour, social standing have the potential to affect *Strongyloides* transmission within baboons, however these are typically understudied. Demonstrating this, only two studies were found to attempt investigation into temporal factors in this review, however Pettifer was unable to make comparisons due to low sample size [66, 88]. Ebbert et al. [66] showed a 12-fold decline in *Strongyloides* prevalence between 1978 and 2000, yet no explanation was given. Due to the high use of single-point, cross-sectional studies the effect of these factors remains unclear in many localities. An increase in longitudinal studies would allow for a more holistic understanding as significant trends could be tracked over time.

Investigations show no significant difference between *Strongyloides* infections in males and female baboons however age does appear to play a role [83, 88, 90, 92]. Overall, infection prevalence is significantly lower in older animals, perhaps due to acquired immunity with age or reduced larval/egg excretions. Although, finding a higher prevalence in adolescent chacma baboons compared to pre-puberty and adult stages, Pettifer [88] showed a complete lack of *S. fuelleborni* infections within infant, weaner and intermediate-juvenile individuals. This proves somewhat interesting as trans-mammary infection is known to occur in this *Strongyloides* species, however sample size was very small [97].

Social rank can increase the levels of stress hormones in NHPs, especially in terms of dominant individuals, leading to negative health outcomes [98–101]. Within baboons, outcomes are mixed regarding social rank effects on *Strongyloides* infections; however, this is based on only a handful of studies. Müller Graff et al. [83], demonstrated no significant differences between social rank, whilst Akinyi et al. [57], showed a significant increase in overall parasitism within socially isolated females and Hausfater [102], an increase in dominant males. With such limited available data more needs to be done to fully understand infection dynamics within troops to identify high-risk individuals and behaviours for potential transmission into human communities.

### Diagnostic methods used for *Strongyloides* detection

Flotation and sedimentation techniques for the visualisation of threadworm eggs/larvae in faecal material were by far the most utilised approach during this review (84% of articles). Although popular, even in human studies, these methods are highly generalised and may struggle to produce evidence of infection as *Strongyloides* release much fewer eggs than other helminths, as well as running the danger of larvae being maintained in debris plugs during popular modifications such as the formalin-ether concentration technique [103, 104]. Additionally, due to their thin-shelled eggs, *S. fuelleborni* may be susceptible to osmotic pressure resulting in their disintegration, leading to false negatives [105]. On inspection, median *Strongyloides* egg/larval counts here ranged from 0.3 eggs per gram (EPG) to 136 EPG [68, 92]. How spatial proximity to humans affects egg output is not yet understood, with this only being made clear by the lowest EPG found coming from crop-raiding baboons and the highest from baboons sharing a water source with humans and livestock. There are likely multiple factors at play here, highlighting the need for further investigation. Gillespie [106] however argues that as egg output is altered by a variety of factors it should not be used to directly infer infection intensity in primate studies.

Wildly underused during NHP investigations, incubation and copro-culture methods such as the Baermann concentration technique (BCT), agar plate culture (APC), charcoal culture and Harada-Mori can dramatically increase the odds of identifying *Strongyloides* in faecal samples [104, 107]. Of the publications reviewed here, less than a quarter undertook one or more of these methodologies with the specific purpose of identifying filiform larvae, of which the Harada-Mori technique was most favoured. Although popular, Harada-Mori has been shown to have weaker sensitivity than BCT and APC, further contributing to the potential underdiagnosis of infection [108]. To note, in this review after excluding flotation/sedimentation methods, *S. stercoralis* was exclusively identified using a BTC and *S. fuelleborni* when either a charcoal culture or Harada-Mori was performed. This alludes to differences in life cycle between these two species as *S. stercoralis* sheds larvae in faeces, whilst *S. fuelleborni* sheds eggs, requiring an extra period of development in culture. The general underrepresentation of culture techniques seen here largely stems from the non-specific approach commonly applied to studies of gastrointestinal parasites in both NHPs and humans alike. Although requiring more time, resources and the incubation of potentially infective material, cultures do allow for increased sensitivity as well as the isolation of larvae from faecal material which can then be used for improved morphological identification, molecular testing and sequencing.

### Molecular epidemiology

Whilst a powerful diagnostic tool, the use of molecular DNA based identification of *Strongyloides* is nascent with less than 7% of studies analysed here utilising either PCR or sequencing methods [15, 43, 44]. Despite their underuse, real time-PCRs are available to identify *Strongyloides* from faecal material using the 18S rDNA gene, achieving 100% genus-level specificity and high sensitivity [109]. Nevertheless, this assay cannot discriminate between *S. stercoralis* and *S. fuelleborni* therefore new methodology described by Cunningham et al. [10] has increased utility as it can better discriminate the two species. This method implicated *S. fuelleborni* in ~ 17% of human samples previously identified as positive for *Strongyloides* (assumed *S. stercoralis*) using the previous methodology, highlighting the lack of appreciation of *S. fuelleborni* infections in humans. Encouragingly during optimisation, this assay was also successfully tested on baboon samples collected during investigations by Juhász et al. [44] to identify *S. fuelleborni*, however results are yet to be published. Although an important development, this species-specific assay has reduced sensitivity, therefore a combination of the two methods is recommended.

Sequencing genetic material provides us our only reliable method for accurate species differentiation, genetic sub-typing, linking human-NHP infections and even deciphering host specificity [110]. The use of hyper-variable regions (HVR I and IV) of the 18S rDNA was first introduced by Hasegawa et al. in 2009 and was subsequently used by both Juhász et al. and Anderson et al. during their investigations as sequences are known to be highly discriminatory between *Strongyloides* species [43, 44, 111]. Other targets used for sequence analysis in baboon infections were the internal transcribed spacer regions (ITS 1 and 2) of ribosomal DNA and the mitochondrial loci of cytochrome oxidase I (*cox-1*) [15]. The ITS 1 and 2 have lower levels of intra-specific polymorphisms and are a common choice when discriminating nematodes, whereas *cox-1* is subject to higher rates of mutations making it more amenable for identifying changes between closely related individuals within the same species [112, 113]. To gain a truly comprehensive picture of this genus’ genetic variation it is important to use these markers in combination, not in isolation.

**Fig. 5:**
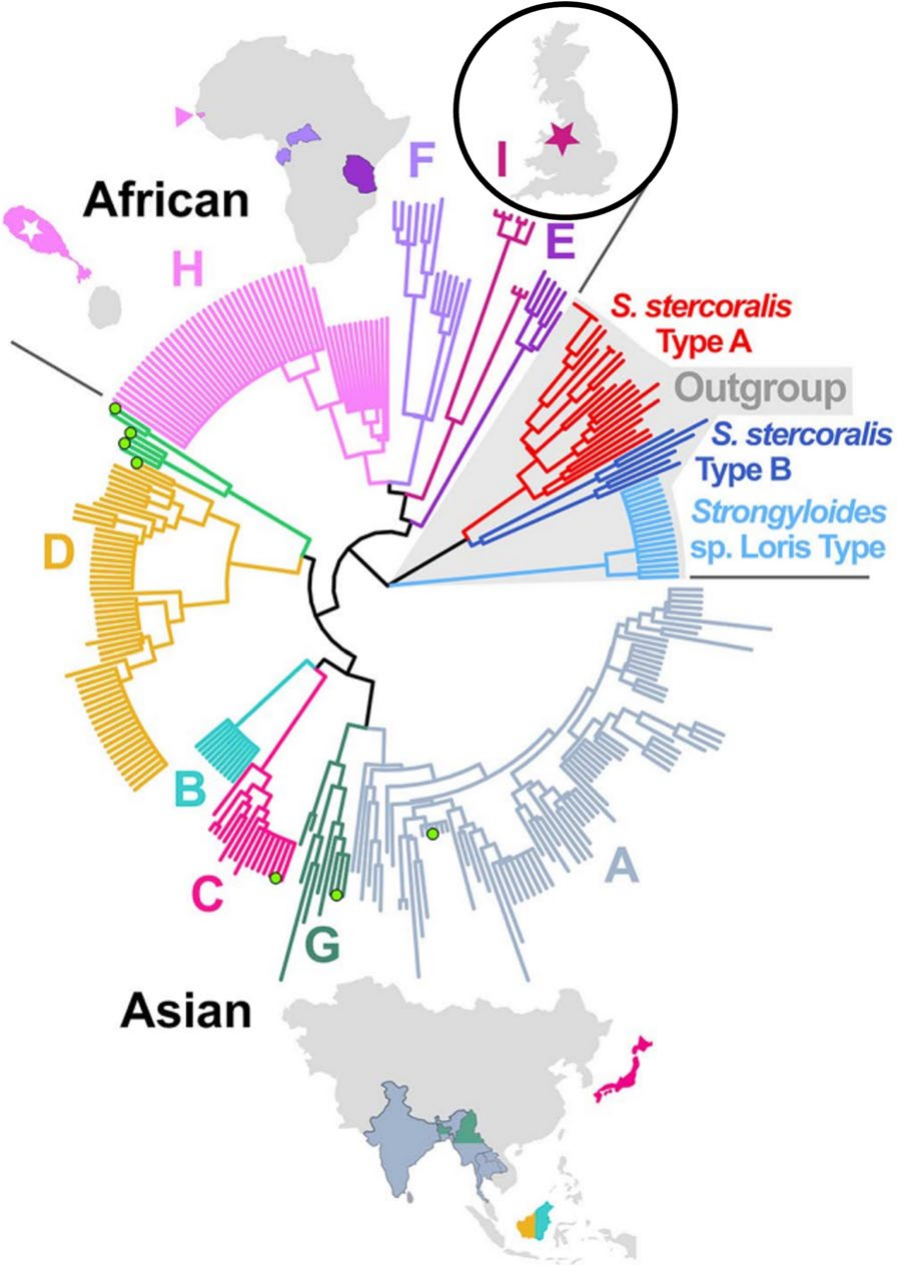
Neighbour-joining phylogenetic tree generated from reference *Strongyloides* genotypes and UK worms (HVR-I, HVR-IV and c*ox-1* sequences), taken and minorly adapted from Richins et al. [31]. The Asian and African *S. fuelleborni* division is demonstrated, as well as the high level of variation found at the UK safari park (cluster I), highlighted by black circle

Recent investigations by Richins et al. [31] clearly demonstrated the wide genetic diversity found within *S. fuelleborni* even within a small geographical area, having undertaken whole genome sequencing, then further focussing on *cox-1* and 18S rDNA loci. Using larval and adult stages of worms extracted from hybrid baboon faecal samples, located within a UK safari park, they were able to show clear African and Asian lineages, a new 18S rDNA HVR IV haplotype and extended intergenic spacer regions of the mitochondrial genome when compared to other published sequences. The infections within this UK baboon troop are also notable due to the high degree of genetic variability shown within the worms from the one confined locality, as seen in Fig. 5, represented by the letter I. One explanation for this high level of diversity is the mixed ancestry of this particular baboon troop, having potentially led to the introgression of two or more *Strongyloides* sub-groups from the varied background of the troop’s predecessors. The degree to which sub-groups introgress on a wider scale is poorly understood and what this might mean for infection and evolutionary dynamics warrants further investigation.

### Anthropogenic effects on baboon parasitism

In terms of anthropogenic factors, Mason et al. [80] showed decreased prevalence within baboons in areas of higher anthropogenic disturbance. Other studies have also shown decreased or similar levels of gastro-intestinal parasitism in baboons engaging in crop foraging, as an alternative to wild foraging [67, 87, 92]. Although this certainly doesn’t exclude zoonotic transmission, it at least demonstrates closer relationships between humans and NHPs may not necessarily lead to increased zoonotic risk. Nevertheless, studies have shown increased *Strongyloides* infections within macaques (a close baboon relative) living in close proximity to humans, therefore it is not unreasonable to assume similar outcomes could also occur in baboons [114, 115]. Again, assessing this on a local level is key as baboons and humans will display different behaviours and levels of interaction at individual locations.

### Zoonotic transmission of *Strongyloides* from baboons

The zoonotic and One Health implications of gastrointestinal parasites in general is mentioned in the vast majority of papers reviewed here, however very few attempt to directly compare infections in sympatric human-baboon populations. Alqumber [58, 59] however has twice discovered *Strongyloides* infections within humans and baboons living in and around cities in Saudi Arabia, with higher prevalences in people living in the city peripheries. It is suggested this increase in prevalence is possibly linked to the higher occurrence of hamadryas baboons in those areas of the city. *Strongyloides fuelleborni* has also been identified within humans and vervets (*Chlorocebus pygerythrus*) living in similar locals in Southern Malawi [116]. Encouragingly however, Kebede et al. [74] demonstrated a complete absence of *Strongyloides* in human Ethiopian populations living in the same area as infected baboons, so it appears its presence in a geographical region may not always give rise to zoonotic transmission.

Even fewer studies attempt to directly genetically link zoonotic infections between baboons to humans. Of these, only one focused on *Strongyloides* specifically, where HVR-IV and *cox-1* sequences were matched in samples from a Japanese mammologist and baboons circulating in the same area [19]. Studies linking zoonotic transmission in any primates are extremely rare, however *Strongyloides* sequences have been matched between humans and macaques in Thailand as well as chimpanzees in Uganda [12, 117, 118]. It is therefore not unreasonable to assume transmission between baboons and humans is highly probable in multiple areas, especially given their resilience to anthropogenic change and that additional research is needed to uncover it. With the uptake of a One Health approach and molecular methods, contributing to the number of genetic sequences available, this situation will inevitably become clearer in time.

Despite acknowledging the zoonotic potential of strongyloidiasis and other helminth infections, little has been achieved to devise appropriate plans for its control. In terms of zoological and research organisations, individuals can be diagnosed and targeted for treatment, upon a definitive diagnosis and/or pathology of threadworms. However, treating wild baboon populations with anthelmintics would likely have multiple ethical, financial and environmental issues [119]. It is also likely to pose significant logistical challenges and would increase the danger of selection and introducing putative drug resistance into areas where control in human populations is paramount [120]. Zoonotic control should therefore favour more practical spatial deterrents for keeping baboons away from areas of high anthropogenic activity via the use of rangers, predator models, bioacoustics (such as bees) and electric fences [121, 122].

## Conclusions

Considering transmission from NHPs into human populations, the zoonotic capabilities of *Strongyloides* spp. remain poorly understood. In Africa, baboons are of special interest as they widespread across the continent, largely terrestrial, resistant to anthropogenic change and are susceptible to the two main species of *Strongyloides* found in humans. Here we judge that infection prevalence in baboons was not affected by infection setting or *Strongyloides* species however a significant differences in infection prevalence was found between *P. cynocephalus* and *P. papio*, with little cross species studies to draw unequivocal deductions. Overall, a distinct lack of molecular methods applied in the diagnosis of strongyloidiasis ultimately hampers our understanding of species dynamics, genetic diversity and host specificity within the *Strongyloides* genus. An increased uptake of molecular methods for the identification of *Strongyloides* infections is recommended, allowing for better infection tracking between human and NHP populations via DNA sequence comparisons and speciation. As with many gastrointestinal helminths, the consideration of reservoir hosts is paramount to ensure control measures consider zoonotic transmission, giving rise to improved disease monitoring and outcomes for all.

## Supplementary Information

**Additional file 1**. Database search terms.

## Data Availability

All data analysed during this study is taken from articles outlined in Table 2.

## Abbreviations

NHP: Non-human primate
STH: Soil-transmitted helminth
WHO: World Health Organization
SBS: Swollen belly syndrome
IQR: Inter-quartile range
ND: Not defined
EPG: Eggs per gram
BCT: Baermann concentration technique
APC: Agar plate culture
rt-PCR: Real-time polymerase chain reaction
HVR: Hyper-variable region
ITS: Internal transcribed spacer
*Cox-1*: Cytochrome oxidase 1

## References

[1] Perelman P, Johnson WE, Roos C, Seuanez HN, Horvath JE, Moreira MA, et al. A molecular phylogeny of living primates. PLoS Genet. 2011;7(3):e1001342. 21436896 10.1371/journal.pgen.1001342 PMC3060065

[2] Lappan S, Malaivijitnond S, Radhakrishna S, Riley EP, Ruppert N. The human-primate interface in the new normal: challenges and opportunities for primatologists in the COVID-19 era and beyond. Am J Primatol. 2020;82(8):e23176. 32686188 10.1002/ajp.23176 PMC7404331

[3] Cascio A, Bosilkovski M, Rodriguez-Morales AJ, Pappas G. The socio-ecology of zoonotic infections. Clin Microbiol Infect. 2011;17(3):336–42. 21175957 10.1111/j.1469-0691.2010.03451.x

[4] Ofori BY, Annan AB, Asomaning B, Vigbedor DT, Asante PA. Sharing without caring: High prevalence and similarity of potentially zoonotic gastrointestinal helminths in two sympatric nonhuman primates in a tropical resource reserve. Int J Ecol. 2024;2024:6081533.

[5] WHO. Ending the neglect to attain the sustainable development goals: a road map for neglected tropical diseases 2021–2030. Geneva: World Health Organization; 2020.

[6] Olsen A, van Lieshout L, Marti H, Polderman T, Polman K, Steinmann P, et al. Strongyloidiasis - the most neglected of the neglected tropical diseases? Trans R Soc Trop Med Hyg. 2009;103(10):967–72. 19328508 10.1016/j.trstmh.2009.02.013

[7] Buonfrate D, Bisanzio D, Giorli G, Odermatt P, Furst T, Greenaway C, et al. The global prevalence of *Strongyloides stercoralis* infection. Pathogens. 2020;9(6):468. 32545787 10.3390/pathogens9060468 PMC7349647

[8] Nosková E, Sambucci KM, Petrž Elková KJ, Červená B, Modrý D, Pafčo B. *Strongyloides* in non-human primates: significance for public health control. Philos Trans R Soc Lond B Biol Sci. 2024;379:20230006(1894). 10.1098/rstb.2023.0006 PMC10676817 38008123

[9] Toledo R, Munoz-Antoli C, Esteban JG. Strongyloidiasis with emphasis on human infections and its different clinical forms. Adv Parasitol. 2015;88:165–241. 25911368 10.1016/bs.apar.2015.02.005

[10] Cunningham LJ, Nevin WD, Verweij JJ, Buonfrate D, Scarso S, Khieu V, et al. Improving molecular epidemiological surveillance of strongyloidiasis upon differentiation of *Strongyloides fuelleborni fuelleborni* from *Strongyloides stercoralis*. J Infect Dis. 2025;232:e169–73. 40423557 10.1093/infdis/jiaf237 PMC12308652

[11] Hasegawa H, Sato H, Fujita S, Mbehang-Nguema PPM, Nobusue K, Miyagi K, et al. Molecular identification of the causative agent of human strongyloidiasis acquired in Tanzania: dispersal and diversity of *Strongyloides* spp. and their hosts. Parasit Int. 2010;59(3):407–13. 10.1016/j.parint.2010.05.007 20621633

[12] Janwan P, Rodpai R, Intapan PM, Sanpool O, Tourtip S, Maleewong W, et al. Possible transmission of *Strongyloides fuelleborni* between working Southern pig-tailed macaques (*Macaca nemestrina*) and their owners in Southern Thailand: Molecular identification and diversity. Infect Genet Evol. 2020;85:104516. 32860989 10.1016/j.meegid.2020.104516

[13] Goldsmid JM. The use of mebendazole as a broad spectrum anthelmintic in Rhodesia. S Afr Med J. 1974;48(54):2265–6. 4432180

[14] Appleton CC, Henzi SP, Whitehead SI. Gastro-intestinal helminth parasites of the chacma baboon, *Papio cynocephalus ursinus*, from the coastal lowlands of Zululand, South Africa. Afr J Ecol. 1991;29(2):149–56.

[15] Obanda V, Maingi N, Muchemi G, Ng’Ang’A CJ, Angelone S, Archie EA. Infection dynamics of gastrointestinal helminths in sympatric non-human primates, livestock and wild ruminants in Kenya. PLoS ONE. 2019;14(6):e0217929. 31181093 10.1371/journal.pone.0217929 PMC6557494

[16] Penner LR. Concerning threadworm (*Strongyloides stercoralis*) in Great Apes: Lowland Gorillas (*Gorilla gorilla*) and chimpanzees (*Pan troglodytes*). J Zoo Anim Med. 1981;12(4):128–31.

[17] Harper JS 3rd, Genta RM, Gam A, London WT, Neva FA. Experimental disseminated strongyloidiasis in *Erythrocebus patas*. I. Pathology. Am J Trop Med Hyg. 1984;33(3):431–43. 6731675 10.4269/ajtmh.1984.33.431

[18] Buonfrate D, Requena-Mendez A, Angheben A, Munoz J, Gobbi F, Van Den Ende J, et al. Severe strongyloidiasis: a systematic review of case reports. BMC Infect Dis. 2013;13:78. 23394259 10.1186/1471-2334-13-78 PMC3598958

[19] Hasegawa H, Sato H, Fujita S, Mbehang-Nguema PPM, Nobusue K, Miyagi K, et al. Molecular identification of the causative agent of human strongyloidiasis acquired in Tanzania: dispersal and diversity of *Strongyloides* spp. and their hosts. Parasitol Int. 2010;59(3):407–13. 20621633 10.1016/j.parint.2010.05.007

[20] Zhao H, Haidamak J, Noskova E, Ilik V, Pafčo B, Ford R, et al. Insights into infant strongyloidiasis. Papua New Guinea Emerg Infect Dis. 2025;31(9):1793–801. 40867023 10.3201/eid3109.241923 PMC12407202

[21] Mahmoud AA. Strongyloidiasis. Clin Infect Dis. 1996;23(5):949–52 (quiz 53). 8922784 10.1093/clinids/23.5.949

[22] Viney M. *Strongyloides*. Parasitology. 2017;144(3):259–62. 27759560 10.1017/S0031182016001773 PMC5364833

[23] Farthing M, Albonico M, Bisoffi Z, Bundy D, Buonfrate D, Chiodini P, et al. World gastroenterology organisation global guidelines: management of strongyloidiasis: February 2018—compact version. J Clin Gastroenterol. 2020;54(9):747–57. 32890112 10.1097/MCG.0000000000001369

[24] WHO. WHO guideline on preventive chemotherapy for public health control of strongyloidiasis. WHO guidelines approved by the guidelines review committee. Geneva: World Health Organisation; 2024. 39190728

[25] Kagira JM, Oluoch G, Waititu K, Mulei I, Maingi N, Ngotho M. High efficacy of combined albendazole and ivermectin teatment against gastrointestinal nematodes in vervet monkeys and baboons. Scand J Lab Anim Sci. 2011;38(3):187–93.

[26] Kleinschmidt LM, Kinney ME, Hanley CS. Treatment of disseminated *Strongyloides* spp. infection in an infant Sumatran orangutan (*Pongo abelii*). J Med Primatol. 2018;47(3):201–4. 29493782 10.1111/jmp.12338 PMC7166570

[27] Hiebert K, Gardhouse S, Sarvi J, Herrin B, Miller K, Chelladurai JJ. Identification and treatment of *Strongyloides cebus* in captive ring-tailed lemurs (*Lemur catta*) in the midwestern United States. Vet Parasitol Reg Stud Reports. 2023;39:100839. 36878624 10.1016/j.vprsr.2023.100839

[28] Jaleta TG, Zhou S, Bemm FM, Schar F, Khieu V, Muth S, et al. Different but overlapping populations of *Strongyloides stercoralis* in dogs and humans-dogs as a possible source for zoonotic strongyloidiasis. PLoS Negl Trop Dis. 2017;11(8):e0005752. 28793306 10.1371/journal.pntd.0005752 PMC5565190

[29] Barratt JLN, Sapp SGH. Machine learning-based analyses support the existence of species complexes for *Strongyloides fuelleborni* and *Strongyloides stercoralis*. Parasitology. 2020;147(11):1184–95. 32539880 10.1017/S0031182020000979 PMC7443747

[30] Barratt JLN, Lane M, Talundzic E, Richins T, Robertson G, Formenti F, et al. A global genotyping survey of *Strongyloides stercoralis* and *Strongyloides fuelleborni* using deep amplicon sequencing. PLoS Negl Trop Dis. 2019;13(9):26. 10.1371/journal.pntd.0007609 PMC6762204 31525192

[31] Richins T, Sapp SGH, Juhàsz A, Cunningham LJ, La Course EJ, Stothard JR, et al. Genetic diversity within *Strongyloides fuelleborni:* mitochondrial genome analysis reveals a clear African and Asian division. Parasitology. 2025. 10.1017/s0031182025100243. 40545470 10.1017/S0031182025100243 PMC12418279

[32] Alberts SC, Altmann J. Immigration and hybridization patterns of yellow and anubis baboons in and around Amboseli, Kenya. Am J Primatol. 2001;53(4):139–54. 11283975 10.1002/ajp.1

[33] Keller C, Roos C, Groeneveld LF, Fischer J, Zinner D. Introgressive hybridization in Southern African baboons shapes patterns of mtDNA variation. Am J Phys Anthropol. 2010;142(1):125–36. 19918986 10.1002/ajpa.21209

[34] Zinner D, Wertheimer J, Liedigk R, Groeneveld LF, Roos C. Baboon phylogeny as inferred from complete mitochondrial genomes. Am J Phys Anthropol. 2013;150(1):133–40. 23180628 10.1002/ajpa.22185 PMC3572579

[35] Fischer J, Zinner D. Introduction to special issue: frontiers in baboon research. J Hum Evol. 2020;146:102822. 32750559 10.1016/j.jhevol.2020.102822

[36] Jolly CJ. Species, species concepts, and primate evolution. New York: Plenum Press; 1993.

[37] Nature IUfCo. IUCN red list of threatened species [cited 2026 January]. https://www.iucnredlist.org/

[38] Hurn S. Baboon cosmopolitanism: more-than-human moralities in a multispecies community. London: Palgrave Macmillan; 2015. p. 152–66.

[39] Kyokuhaire AM, Chapman CA, Omeja PA, Tumusiime DM, Abwoli BY, Lawes MJ. Mitigating crop raiding by forest elephants and baboons at Kibale National Park. Afr J Ecol. 2023;61(1):129–40.

[40] Mazué F, Guerbois C, Fritz H, Rebout N, Petit O. Less bins, less baboons: reducing access to anthropogenic food effectively decreases the urban foraging behavior of a troop of chacma baboons (*Papio hamadryas ursinus*) in a peri-urban area. Primates. 2023;64(1):91–103. 36436178 10.1007/s10329-022-01032-x

[41] Kifle Z, Bekele A. Human–hamadryas baboon (*Papio hamadryas*) conflict in the Wonchit Valley, South Wollo, Ethiopia. Afr J Ecol. 2021;59(1):29–36.

[42] Leith DA, Mpofu BS, van Velden JL, Reed CC, van Boom KM, Breed D, et al. Are Cape Peninsula baboons raiding their way to obesity and type II diabetes? A comparative study. Comp Biochem Physiol A Mol Integr Physiol. 2020;250:110794. 32827764 10.1016/j.cbpa.2020.110794

[43] Anderson J, Upadhayay R, Sudimack D, Nair S, Leland M, Williams JT, et al. *Trichuris* sp. and *Strongyloides* sp. infections in a free-ranging baboon colony. J Parasitol. 2012;98(1):205–8. 21830937 10.1645/GE-2493.1 PMC3741105

[44] Juhàsz A, Spiers E, Tinsley E, Chapman E, Shaw W, Head M, et al. Gastrointestinal parasites in captive olive baboons in a UK safari park. Parasitology. 2023;150(12):1096–104. 37655745 10.1017/S0031182023000823 PMC10801365

[45] Rivero J, Callejon R, Garcia-Sanchez AM. *Trichuris* infection in captive non-human primates in zoological gardens in Spain. J Helminthol. 2025;99:e1. 39803677 10.1017/S0022149X24000774

[46] Lankau EW, Turner PV, Mullan RJ, Galland GG. Worker health and safety practices in research facilities using nonhuman primates, North America. Emerg Infect Dis. 2014;20(9):1589–90. 25153090 10.3201/eid2009.140420 PMC4178409

[47] Roberts JA. Occupational health concerns with nonhuman primates in zoological gardens. J Zoo Wildl Med. 1995;26(1):10–23.

[48] Cowlishaw G. Behavioural patterns in baboon group encounters: the role of resource competition and male reproductive strategies. Behaviour. 1995;132(1/2):75–86.

[49] Alberts SC. Social influences on survival and reproduction: insights from a long-term study of wild baboons. J Anim Ecol. 2019;88(1):47–66. 30033518 10.1111/1365-2656.12887 PMC6340732

[50] Rogers J, Raveendran M, Harris RA, Mailund T, Leppälä K, Athanasiadis G, et al. The comparative genomics and complex population history of *Papio* baboons. Sci Adv. 2019;5(1):eaau6947. 30854422 10.1126/sciadv.aau6947 PMC6401983

[51] Sørensen EF, Harris RA, Zhang L, Raveendran M, Kuderna LFK, Walker JA, et al. Genome-wide coancestry reveals details of ancient and recent male-driven reticulation in baboons. Science. 2023;380(6648):eabn8153. 37262153 10.1126/science.abn8153

[52] Programme CAS. CASP: cross-sectional checklist 2024. https://casp-uk.net/casp-checklists/CASP-checklist-cross-sectional-study-2024.pdf

[53] Team RC. R: a language and environment for statistical computing. R Foundation for Statistical Computing; 2021.

[54] Dinno A. _dunn.test: Dunn's test of multiple comparisons using rank sums_. R package version 136. 2024.

[55] Abbott DP, Majeed SK. A survey of parasitic lesions in wild-caught, laboratory-maintained primates: (Rhesus, Cynomolgus, and Baboon). Vet Pathol. 1984;21(2):198–207. 6730203 10.1177/030098588402100212

[56] Adeola AJ, Adeola AN, Fajobi EA, Babatunde KO, Akande OA, Ajayi SR. Comparative study of gastrointestinal parasites from captive and wild olive baboon (*Papio anubis*). Niger J Parasitol. 2019;40(2):240–4.

[57] Akinyi MY, Jansen D, Habig B, Gesquiere LR, Alberts SC, Archie EA. Costs and drivers of helminth parasite infection in wild female baboons. J Anim Ecol. 2019;88(7):1029–43. 30972751 10.1111/1365-2656.12994 PMC6957333

[58] Alqumber MA. Association between *Papio hamadryas* populations and human gastrointestinal infectious diseases in southwestern Saudi Arabia. Ann Saudi Med. 2014;34(4):297–301. 25811201 10.5144/0256-4947.2014.297 PMC6152561

[59] Alqumber MA. Microbiological ecological surveillance of zoonotic pathogens from hamadryas baboons in Southwestern Saudi Arabia. Microorganisms. 2024;12(12):12. 10.3390/microorganisms12122421 PMC11677152 39770623

[60] Appleton CC, Henzi SP. Environmental correlates of gastrointestinal parasitism in montane and lowland baboons in Natal, South Africa. Int J Primatol. 1993;14(4):623–35.

[61] Appleton CC, Brain C. Gastro-intestinal parasites of *Papio cynocephalus ursinus* living in the central Namib Desert, Namibia. Afr J Ecol. 1995;33(3):257–65.

[62] Barelli C, Donati C, Albanese D, Pafčo B, Modrý D, Rovero F, et al. Interactions between parasitic helminths and gut microbiota in wild tropical primates from intact and fragmented habitats. 2021. Sci Rep. 10.1038/s41598-021-01145-1. 10.1038/s41598-021-01145-1 PMC8566450 34732823

[63] Barelli C, Pafco B, Manica M, Rovero F, Rosà R, Modry D, et al. Loss of protozoan and metazoan intestinal symbiont biodiversity in wild primates living in unprotected forests. Sci Rep. 2020;10(1):12. 32616818 10.1038/s41598-020-67959-7 PMC7331812

[64] Bezjian M, Gillespie TR, Chapman CA, Greiner EC. Coprologic evidence of gastrointestinal helminths of forest baboons, *Papio anubis*, in Kibale National Park, Uganda. J Wildl Dis. 2008;44(4):878–87. 18957644 10.7589/0090-3558-44.4.878

[65] Bradbury RS, Olson AR, Sapp S, Panicker IS, Foster-Nyarko E, Qvarnstrom Y, et al. Intestinal parasite infection in non-human primates from The Gambia, West Africa, and their relationship to human activity. Parasitology. 2025. 10.1017/s0031182025000514. 40207532 10.1017/S0031182025000514 PMC12186559

[66] Ebbert MA, McGrew WC, Marchant LF. Community composition, correlations among taxa, prevalence, and richness in gastrointestinal parasites of baboons in Senegal, West Africa. Primates. 2013;54(2):183–9. 23271438 10.1007/s10329-012-0339-x

[67] Eley RM, Strum SC, Muchemi G, Reid GDF. Nutrition, body condition, activity patterns, and parasitism of free‐ranging troops of olive baboons (*Papio anubis*) in Kenya. Am J Primatol. 1989;18(3):209–19. 31964035 10.1002/ajp.1350180304

[68] Fagiolini M, Lia RP, Laricchiuta P, Cavicchio P, Mannella R, Cafarchia C, et al. Gastrointestinal parasites in mammals of two Italian zoological gardens. J Zoo Wildl Med. 2010;41(4):662–70. 21370648 10.1638/2010-0049.1

[69] Fredrick M, Danson M, John K, Stanislaus K, David N, Maina N, et al. *Schistosoma mansoni* and soil transmtted helminths in olive baboons and potential zoonosis. Vet med sci. 2021;7(5):2026–31. 33942545 10.1002/vms3.495 PMC8464276

[70] Habig B, Chowdhury S, Monfort SL, Brown JL, Swedell L, Foerster S. Predictors of helminth parasite infection in female chacma baboons (*Papio ursinus*). Int J Parasitol Parasites Wildl. 2021;14:308–20. 33898232 10.1016/j.ijppaw.2021.03.012 PMC8056146

[71] Hahn NE, Proulx D, Muruthi PM, Alberts S, Altmann J. Gastrointestinal parasites in free-ranging Kenyan baboons (*Papio cynocephalus* and *P. anubis*). Int J Primatol. 2003;24(2):271–9.

[72] Hope K, Goldsmith ML, Graczyk T. Parasitic health of olive baboons in Bwindi Impenetrable National Park, Uganda. Vet Parasitol. 2004;122(2):165–70. 15177721 10.1016/j.vetpar.2004.03.017

[73] Howells ME, Pruetz J, Gillespie TR. Patterns of gastro-intestinal parasites and commensals as an index of population and ecosystem health: the case of sympatric western chimpanzees (*Pan troglodytes verus*) and guinea baboons (*Papio hamadryas papio*) at Fongoli, Senegal. Am J Primatol. 2011;73(2):173–9. 20853397 10.1002/ajp.20884

[74] Kebede T, Negash Y, Erko B. *Schistosoma mansoni* infection in human and nonhuman primates in selected areas of Oromia Regional State, Ethiopia. J Vector Borne Dis. 2018;55(2):116–21. 30280709 10.4103/0972-9062.242558

[75] Kooriyama T, Hasegawa H, Shimozuru M, Tsubota T, Nishida T, Iwaki T. Parasitology of five primates in Mahale Mountains National Park, Tanzania. Primates. 2012;53(4):365–75. 22661394 10.1007/s10329-012-0311-9

[76] Larbi JA, Akyeampong S, Abubakari A, Offei Addo S, Okoto D, Hanson H. Zoonotic gastrointestinal parasites of baboons (*Papio anubis*) in the Shai Hill Reserve in Ghana. Biomed Res Int. 2020;2020:1083251. 32258100 10.1155/2020/1083251 PMC7086414

[77] Larbi JA, Akyeampong S, Addo SO, Dakwa KB, Boampong K, Opoku-Nketiah B. Distribution of intestinal parasites of baboons (*Papio anubis*) and warthogs (*Phacochoerus aethiopicus*) at the Mole National Park, Ghana. Vet Med Sci. 2021;7(1):251–5. 32772510 10.1002/vms3.335 PMC7840207

[78] Legesse M, Erko B. Zoonotic intestinal parasites in *Papio anubis* (baboon) and *Cercopithecus aethiops* (vervet) from four localities in Ethiopia. Acta Trop. 2004;90(3):231–6. 15099809 10.1016/j.actatropica.2003.12.003

[79] Mafuyai HB, Barshep Y, Audu BS, Kumbak D, Ojobe TO. Baboons as potential reservoirs of zoonotic gastrointestinal parasite infections at Yankari National Park, Nigeria. Afr Health Sci. 2013;13(2):252–4. 24235920 10.4314/ahs.v13i2.7 PMC3824496

[80] Mason B, Piel AK, Modry D, Petrzelkova KJ, Stewart FA, Pafao B. Association of human disturbance and gastrointestinal parasite infection of yellow baboons in western Tanzania. PLoS ONE. 2022;17(1):19. 10.1371/journal.pone.0262481 PMC8754341 35020760

[81] Mbuthia P, Murungi E, Owino V, Akinyi M, Eastwood G, Nyamota R, et al. Potentially zoonotic gastrointestinal nematodes co-infecting free ranging non-human primates in Kenyan urban centres. Vet Med Sci. 2021;7(3):1023–33. 33400394 10.1002/vms3.424 PMC8136933

[82] McGrew WC, Tutin CEG, Collins DA, File SK. Intestinal parasites of sympatric *Pan troglodytes* and *Papio* spp. at two sites: Gombe (Tanzania) and Mt. Assirik (Senegal). Am J Primatol. 1989;17(2):147–55. 31968849 10.1002/ajp.1350170204

[83] Müller-Graf CDM, Collins DA, Woolhouse MEJ. Intestinal parasite burden in five troops of olive baboons (*Papio cynocephalus anubis*) in Gombe Stream National Park, Tanzania. Parasitology. 1996;112:489–97. 8677138 10.1017/s0031182000076952

[84] Munene E, Otsyula M, Mbaabu DAN, Mutahi WT, Muriuki SMK, Muchemi GM. Helminth and protozoan gastrointestinal tract parasites in captive and wild-trapped African non-human primates. Vet Pathol. 1998;78(3):195–201. 10.1016/s0304-4017(98)00143-5 9760061

[85] Ocaido M, Dranzoa C, Cheli P. Gastrointestinal parasites of baboons (*Papio anubis*) interacting with humans in West Bugwe Forest Reserve, Uganda. Afr J Ecol. 2003;41(4):356–9.

[86] Owen D, Casillo S. A preliminary survey of the nematode parasites of some imported old-world monkeys. Lab Anim. 1973;7(3):265–9. 4201086 10.1258/002367773780944120

[87] Pebsworth PA, Archer C, Appleton CC, Huffman MA. Parasite transmission risk from geophagic and foraging behavior in chacma baboons. Am J Primatol. 2012;74(10):940–7. 22707091 10.1002/ajp.22046

[88] Pettifer HL. The helminth fauna of the digestive tracts of chacma baboons, *Papio ursinus*, from different localities in the Transvaal. Onderstepoort J Vet Res. 1984;51(3):161–70. 6533507

[89] Reichard MV, Thomas JE, Chavez-Suarez M, Cullin CO, White GL, Wydysh EC, et al. Pilot Study to assess the efficacy of ivermectin and fenbendazole for treating captive-born olive baboons (*Papio anubis*) coinfected with *Strongyloides fülleborni* and *Trichuris trichiura*. J Am Assoc Lab Anim Sci. 2017;56(1):52–6. 28905715 PMC5250495

[90] Ryan SJ, Brashares JS, Walsh C, Milbers K, Kilroy C, Chapman CA. A survey of gastrointestinal parasites of olive baboons (*Papio anubis*) in human settlement areas of Mole National Park, Ghana. J Parasitol. 2012;98(4):885–8. 22300265 10.1645/GE-2976.1

[91] Shemshadi B, Ranjbar-Bahadori S, Jahani S. Prevalence and intensity of intestinal helminths in carnivores and primates at Vakilabad Zoo in Mashhad, Iran. Comp Clin Pathol. 2015;24(2):387–91.

[92] Weyher AH, Ross C, Semple S. Gastrointestinal parasites in crop raiding and wild foraging *Papio anubis* in Nigeria. Int J Primatol. 2006;27(6):1519–34.

[93] Fehlmann G, O’Riain MJ, Kerr-Smith C, Hailes S, Luckman A, Shepard ELC, et al. Extreme behavioural shifts by baboons exploiting risky, resource-rich, human-modified environments. Sci Rep. 2017;7(1):15057. 29118405 10.1038/s41598-017-14871-2 PMC5678166

[94] Murray S, Stem C, Boudreau B, Goodall J. Intestinal parasites of baboons (*Papio cynocephalus anubis)* and chimpanzees (*Pan troglodytes*) in Gombe National Park. J Zoo Wildl Med. 2000;31(2):176–8. 10982128 10.1638/1042-7260(2000)031[0176:IPOBPC]2.0.CO;2

[95] Vonfeld I, Prenant T, Polack B, Guillot J, Quintard B. Gastrointestinal parasites in non-human primates in zoological institutions in France. Parasite. 2022. 10.1051/parasite/2022040. 36125313 10.1051/parasite/2022040 PMC9487514

[96] Sangpeng J, Eamudomkarn C, Hongsrichan N, Artchayasawat A, Chaisongkram C, Ponsrila K, et al. Prevalence of gastrointestinal parasites in captive mammals at Khon Kaen Zoo, Thailand. Vet World. 2023;16(12):2416–24. 38328364 10.14202/vetworld.2023.2416-2424 PMC10844781

[97] Brown RC, Girardeau HF. Transmammary passage of *Strongyloides* spp. larvae in the human host. Am J Trop Med Hyg. 1977;26(2):215–9. 848643 10.4269/ajtmh.1977.26.215

[98] Gesquiere LR, Learn NH, Simao MC, Onyango PO, Alberts SC, Altmann J. Life at the top: rank and stress in wild male baboons. Science. 2011;333(6040):357–60. 21764751 10.1126/science.1207120 PMC3433837

[99] Abbott DH, Keverne EB, Bercovitch FB, Shively CA, Mendoza SP, Saltzman W, et al. Are subordinates always stressed? A comparative analysis of rank differences in cortisol levels among primates. Horm Behav. 2003;43(1):67–82. 12614636 10.1016/s0018-506x(02)00037-5

[100] Muehlenbein MP, Watts DP. The costs of dominance: testosterone, cortisol and intestinal parasites in wild male chimpanzees. Biopsychosoc Med. 2010;4:21. 21143892 10.1186/1751-0759-4-21 PMC3004803

[101] Habig B, Archie EA. Social status, immune response and parasitism in males: a meta-analysis. Philos Trans R Soc Lond B Biol Sci. 2015;370(1669):20140109. 25870395 10.1098/rstb.2014.0109 PMC4410375

[102] Hausfater G. Dominance and reproduction in Baboons (*Papio cynocephalus*). PhD Thesis, University of Chicago; 1974. 1170998

[103] Anamnart W, Pattanawongsa A, Intapan PM, Maleewong W. Factors affecting recovery of *Strongyloides stercoralis* larvae: an approach to a newly modified formalin-ether concentration technique for diagnosis of strongyloidiasis. J Clin Microbiol. 2010;48(1):97–100. 19923489 10.1128/JCM.01613-09 PMC2812279

[104] Hailu T, Amor A, Nibret E, Munshea A, Anegagrie M, Flores-Chavez MD, et al. Evaluation of five diagnostic methods for *Strongyloides stercoralis* infection in Amhara National Regional State, northwest Ethiopia. BMC Infect Dis. 2022;22(1):297. 35346087 10.1186/s12879-022-07299-1 PMC8962492

[105] Steinbaum L, Kwong LH, Ercumen A, Negash MS, Lovely AJ, Njenga SM, et al. Detecting and enumerating soil-transmitted helminth eggs in soil: New method development and results from field testing in Kenya and Bangladesh. PLoS Negl Trop Dis. 2017;11(4):e0005522. 28379956 10.1371/journal.pntd.0005522 PMC5393894

[106] Gillespie TR. Non-invasive assessment of gastro-intestinal parasite infections in free-ranging primates. Int J Primatol. 2006;27:363–4.

[107] Inês EdJ, Souza JN, Santos RC, Souza ES, Santos FL, Silva ML, et al. Efficacy of parasitological methods for the diagnosis of *Strongyloides stercoralis* and hookworm in faecal specimens. Acta Trop. 2011;120(3):206–10. 21896267 10.1016/j.actatropica.2011.08.010

[108] Nieves E, Fleitas P, Juarez M, Almazan C, Flores G, Alani J, et al. Comparison of parasitological methods for the identification of soil-transmitted helminths, including *Strongyloides stercoralis*, in a regional reference laboratory in northwestern Argentina: An observational study. Parasite Epidemiol Control. 2024;26:e00370. 39139793 10.1016/j.parepi.2024.e00370 PMC11321430

[109] Verweij JJ, Canales M, Polman K, Ziem J, Brienen EA, Polderman AM, et al. Molecular diagnosis of *Strongyloides stercoralis* in faecal samples using real-time PCR. Trans R Soc Trop Med Hyg. 2009;103(4):342–6. 19195671 10.1016/j.trstmh.2008.12.001

[110] Bradbury RS, Pafco B, Nosková E, Hasegawa H. *Strongyloides* genotyping: a review of methods and application in public health and population genetics. Int J Parasitol. 2021;51(13–14):1153–66. 34757088 10.1016/j.ijpara.2021.10.001

[111] Hasegawa H, Hayashida S, Ikeda Y, Sato H. Hyper-variable regions in 18S rDNA of *Strongyloides* spp. as markers for species-specific diagnosis. Parasitol Res. 2009;104(4):869–74. 19050926 10.1007/s00436-008-1269-9

[112] Poon RWS, Tam EWT, Lau SKP, Cheng VCC, Yuen KY, Schuster RK, et al. Molecular identification of cestodes and nematodes by *cox1* gene real-time PCR and sequencing. Diagn Microbiol Infect Dis. 2017;89(3):185–90. 28865743 10.1016/j.diagmicrobio.2017.07.012

[113] Blouin MS. Molecular prospecting for cryptic species of nematodes: mitochondrial DNA versus internal transcribed spacer. Int J Parasitol Parasites Wildl. 2002;32(5):527–31. 10.1016/s0020-7519(01)00357-5 11943225

[114] Hussain S, Ram MS, Kumar A, Shivaji S, Umapathy G. Human presence increases parasitic load in endangered lion-tailed macaques (*Macaca silenus*) in its fragmented rainforest habitats in Southern India. PLoS ONE. 2013;8(5):e63685. 23717465 10.1371/journal.pone.0063685 PMC3661510

[115] Wenz-Mücke A, Sithithaworn P, Petney TN, Taraschewski H. Human contact influences the foraging behaviour and parasite community in long-tailed macaques. Parasitology. 2013;140(6):709–18. 23363557 10.1017/S003118201200203X

[116] Juhàsz A, Makaula P, Cunningham LJ, Archer J, Cowlishaw R, Jones S, et al. Notes on the threadworm *Strongyloides fuelleborni* (Nematoda: Strongyloididae) in vervet monkeys (*Chlorocebus pygerythrus*) and zoonotic strongyloidiasis in southern Malawi. Int J Parasitol Parasites Wildl. 2025. 10.1016/j.ijppaw.2025.101121. 40787158 10.1016/j.ijppaw.2025.101121 PMC12332877

[117] Thanchomnang T, Intapan PM, Sanpool O, Rodpai R, Tourtip S, Yahom S, et al. First molecular identification and genetic diversity of *Strongyloides stercoralis* and *Strongyloides fuelleborni* in human communities having contact with long-tailed macaques in Thailand. Parasitol Res. 2017;116(7):1917–23. 28500375 10.1007/s00436-017-5469-z

[118] Hasegawa H, Kalousova B, McLennan MR, Modry D, Profousova-Psenkova I, Shutt-Phillips KA, et al. *Strongyloides* infections of humans and great apes in Dzanga-Sangha Protected Areas, Central African Republic and in degraded forest fragments in Bulindi, Uganda. Parasitol Int. 2016;65(5 Pt A):367–70. 27180094 10.1016/j.parint.2016.05.004

[119] Vokral I, Podlipna R, Matouskova P, Skalova L. Anthelmintics in the environment: their occurrence, fate, and toxicity to non-target organisms. Chemosphere. 2023;345:140446. 37852376 10.1016/j.chemosphere.2023.140446

[120] Fissiha W, Kinde MZ. Anthelmintic resistance and its mechanism: a review. Infect Drug Resist. 2021;14:5403–10. 34938088 10.2147/IDR.S332378 PMC8687516

[121] Findlay LJ, Lucas C, Walker EM, Evers S, Hill RA. Testing the short-term effectiveness of various deterrents for reducing crop foraging by primates. S Afr J Wildl Res. 2022;52(1):29–43.

[122] van Doorn AC, O’Riain MJ. Nonlethal management of baboons on the urban edge of a large metropole. Am J Primatol. 2020;82(8):e23164. 32602204 10.1002/ajp.23164

