# Supplementary material for: Spotlighting zoonotic strongyloidiasis: a semi-systematic review of threadworms within baboons highlights opportunities for human infections
Source: Infect Dis Poverty. 2026 Jun 17;15:70. doi: 10.1186/s40249-026-01459-0 (PMC13274019; doi:10.1186/s40249-026-01459-0)
Supplement: Supplementary file 1 — Additional file 1. Database search terms. [file 40249_2026_1459_MOESM1_ESM.pdf]

Database	Search Terms
PubMed	(baboon* OR Papio OR primate*[Title/Abstract]) AND (strongyloid* OR threadworm* OR gastro-intestinal OR gastrointestinal OR “soil transmitted helminth” OR helminth OR STH[Title/Abstract]).
Scopus	(baboon* OR Papio OR primate*[TITLE-ABS-KEY]) AND (strongyloid* OR threadworm* OR gastro-intestinal OR gastrointestinal OR “soil transmitted helminth” OR helminth OR STH[TITLE-ABS-KEY]).
Web of Science	(baboon* OR Papio OR primate*[Topic]) AND (strongyloid* OR threadworm* OR gastro-intestinal OR gastrointestinal OR “soil transmitted helminth” OR helminth OR STH[Topic]).
